# Ceramic planar waveguide laser of non-aqueous tape casting fabricated YAG/Yb:YAG/YAG

**DOI:** 10.1038/srep31289

**Published:** 2016-08-18

**Authors:** Chao Wang, Wenxue Li, Chao Yang, Dongbi Bai, Jiang Li, Lin Ge, Yubai Pan, Heping Zeng

**Affiliations:** 1 State Key Laboratory of Precision Spectroscopy, East China Normal University, Shanghai 200062, P. R. China; 2 Key Laboratory of Transparent Opto-functional Inorganic Materials, Shanghai Institute of Ceramics, Chinese Academy of Sciences, Shanghai 200050, China

## Abstract

Ceramic YAG/Yb:YAG/YAG planar waveguide lasers were realized on continuous-wave and mode-locked operations. The straight waveguide, fabricated by non-aqueous tape casting and solid state reactive sintering, enabled highly efficient diode-pumped waveguide continuous-wave laser with the slope efficiency of 66% and average output power of more than 3 W. The influence of the waveguide structure on the wavelength tunability was also experimentally investiccgated with a dispersive prism. Passively mode-locked operation of the ceramic waveguide laser was achieved by using a semiconductor saturable absorber mirror (SESAM), output 2.95 ps pulses with maximum power of 385 mW at the central wavelength of 1030 nm.

As planar waveguide structures offer particularities of high gain, spatial mode control, exceptional thermal management, and optical confinement with respect to traditional bulk materials^1^, planar waveguide laser is believed to play a crucial role in attaining efficient and compact lasers of high average powers and low lasing thresholds^2^. Various planar waveguide gain mediums around 1 μm with crystalline and ceramic material have been progressively investigated in the demonstration of both continuous-wave (CW) and Q-switched lasers, such as Yb:KYW^3^, Nd:YAG^4^, Yb:Y_2_O_3_^5^, Yb:Lu_2_O_3_^6^, and Yb:YAG^7^. Among those materials, Yb:YAG has been widely discussed in waveguide lasers due to its excellent properties including wide emission bandwidth, high pump efficiency, and low quantum defects^7^,^8^,^9^. An efficient high-power laser with output power of 400 W was demonstrated in a Yb:YAG planar waveguide fabricated by using an adhesive-free contact bonding technique^7^. An efficient laser performance with a record slope efficiency of 79% has been achieved in a femtosecond laser fabricated Yb:YAG crystal waveguide^8^. Recently, Q-switched operation with a pulse duration of 88 ns was obtained in such channel waveguides by using carbon nanotubes^9^. In contrast to single crystals, Yb:YAG ceramics are highlighted due to superior thermal properties, higher available doping concentrations as well as less fabrication-consuming. Outstanding laser performances have been demonstrated in both CW and pulsed regimes^10^,^11^,^12^ As of yet, Yb:YAG waveguide ceramic are usually fabricated with ultrafast pulses laser^13^,^14^.

As an established ceramic fabrication technology, the tape casting method is an effective way to fabricate multiplayer ceramic for various materials, not only permitting modification of the refractive index of a bulk sample^15^, but also structuring ceramic waveguides with different refractive indices^16^. The thickness of each tape in this technology can be controlled at micron level, which is very suitable for preparing planar waveguide ceramics. Recently, efficient laser performance of a tape casting produced YAG/Nd:YAG/YAG planar waveguide ceramic was demonstrated in both CW and Q-switched regimes, generating CW laser with 840 mW output power and 65% slope efficiency, and stable pulses with pulse duration of 179 ns, respectively^17^. To date, no planar waveguide lasers were demonstrated in non-aqueous tape casting fabricated YAG/Yb:YAG/YAG ceramic.

On the other hand, the design of composite structure was demonstrated useful to manage thermal effects which are the most critical issues in the gain medium^18^,^19^,^20^. The planar waveguide geometry is a special kind of composite structure and allows for efficient one-dimensional heat flow from the active region due to its high width-to-height aspect-ratio, thereby minimizing deleterious thermal effects^21^. Therefore, the combination of planar waveguide and mode-locking techniques allows for generating stable ultrafast pulse laser with high optical efficiency, which can be used in many applications such as ultrafast spectroscopy, metrology, precision material processing, and ultrafast microscopy. In this Letter, we present the experimental results on CW and mode-locked laser actions in a non-aqueous tape casting fabricated YAG/Yb:YAG/YAG planar waveguide ceramic. From the planar waveguide with 100 μm thick inner layer, CW laser with high slope efficiency and mode-locked laser with stable pulses were achieved.

## Sample fabrication

**Figure 1:**
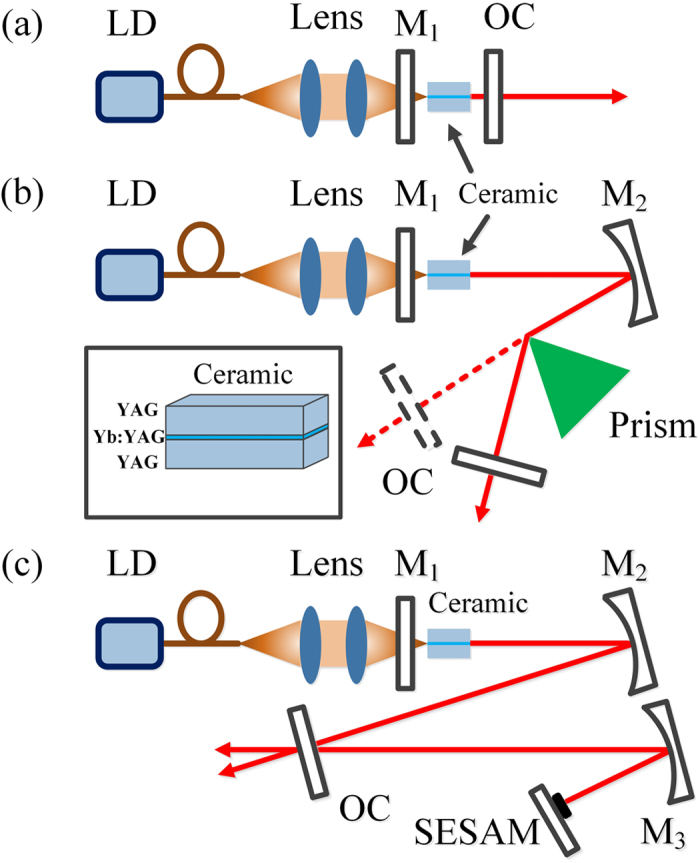
Schematic of YAG/Yb:YAG/YAG ceramic waveguide lasers. (**a**) CW laser operation with plano-plano cavity. (**b**) CW laser operation and wavelength-tuning with three-mirror cavity. (**c**) Mode-locked laser with a SESAM. The inset shows the magnified 3D image of the ceramic. LD, fiber-coupled CW diode laser; M1, end mirror; M2, M3 folding mirrors; OC, output coupler.

The planar waveguide ceramic used in the experiment was fabricated by the combination of non-aqueous tape casting and vacuum sintering method^16^. As shown in the inset of Fig. 1(b), the ceramic was designed with dimension of 3.5 × 3.5 × 3.5 mm^3^, comprising of two 1.7-mm-thick undoped YAG outer layers and 0.1-mm-thick 10 at.% Yb:YAG inner layer with symmetrical arrangement. The refractive indices of the inner and cladding layers were 1.8166 and 1.8154 at 1029 nm, respectively. Since the core-cladding refractive index differences were 1.2 × 10^−3^, the effective waveguides could be easily obtained. Considering the diffusion of Yb^3+^ ions during the sintering process, the total thickness of the part that contained Yb^3+^ ions was about 300 μm. The end faces were optically polished and coated with high transmittance (HT) from 900 nm to 1120 nm.

## Experimental schematic and process

The experimental setup is depicted in Fig. 1A CW 976 nm high-brightness diode laser with a fiber core diameter of 100 μm and a numerical aperture of 0.22 was employed as the end-pumping source. The pump beam was imaged into the ceramic sample with a spot diameter of 100 μm through a telescopic system which consisted of two convex lenses with the same focal length of 50 mm. To keep the laser at a constant temperature for stable and efficient laser operations, the sample was wrapped with indium and mounted in a water-cooled copper heat sink which was maintained at 14 °C during the experiments. The mirror M_1_ with high transmittance for the pump wavelength and high reflectivity (HR) from 1000 nm to 1120 nm served as pump-in coupling and end mirror. The sample was placed close to M_1_ and the distance could be slightly changed for adjustment of mode matching.Firstly, CW laser operation of the YAG/Yb:YAG/YAG planar waveguide ceramic was investigated based on plano-plano and three-mirror laser cavities, respectively. Two output couplers with different transmissions (5% and 10%) from 1000 nm to 1120 nm were utilized in both the cavities to evaluate the laser performances. In the typical setup of plano-plano laser cavity, the output mirror was set close to the waveguide end-face to minimize the diffractive loss. In the three-mirror laser cavity, the concave mirror M_2_ (HT coated at 976 nm, HR coated from 1000 nm to 1120 nm) with curvature radius of 500 mm was placed with small folding angle and 250 mm distance from M_1_ as shown in Fig. 1(b). A Brewster prism was inserted between M_2_ and output coupler in the cavity to investigate the influence of the waveguide structure on the wavelength tunability, and it was placed at Brewster’s angle to decrease the inserted loss. In order to realize the passively mode-locked pulse operation, a SESAM and a concave mirror M_3_ (HT coated at 976 nm, HR coated from 1000 nm to 1120 nm) were added to form a typical Z-fold cavity, as displayed in Fig. 1(c). The curvature radius of M_3_ was selected with 300 mm to focus the laser beam onto the SESAM with appropriate laser fluence. The SESAM with 2% saturation absorption at 1040 nm, 70 μJ/cm^2^ saturation fluence and 500 fs relaxation time constant was mounted on a heat sink without active cooling. In the experiment, we found that, compared to ceramics of conventional structures, the planar waveguide medium was more sensitive to the mode-matching of the cavity and could easily result in a multimode output beam. Thus it’s necessary to design the cavity with appropriate spot beam in the sample, and the length between the end mirror M_1_ and sample had to be optimized for operating lasers in the fundamental mode.

## Results and Discussion

### Characterization of the CW laser operations

**Figure 2:**
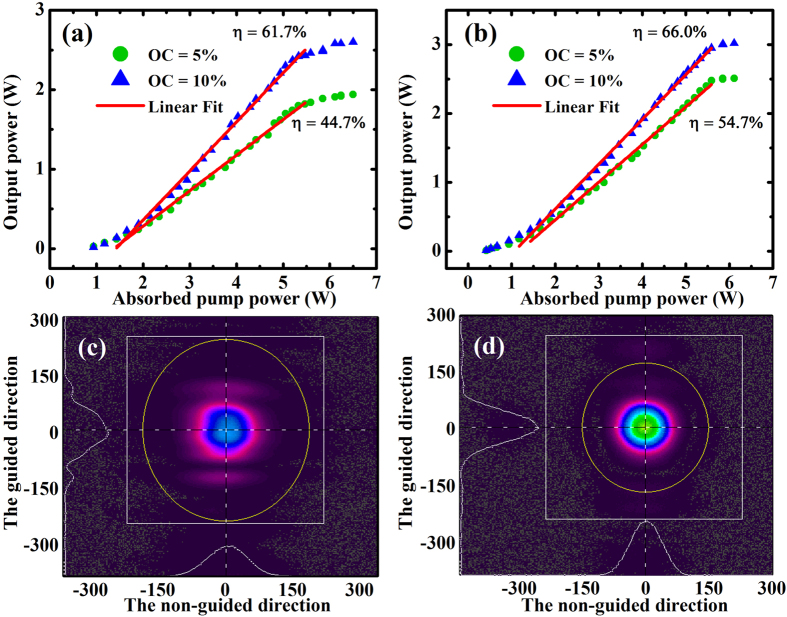
CW laser operations of the YAG/Yb:YAG/YAG planar waveguide ceramic. Average output power characteristics of (**a**) plano-plano laser cavity and (**b**) three-mirror laser cavity with two different output coupler transmissions (5% and 10%). (**c**) The mode profile for the plano-plano laser cavity. (**d**) The mode profile for the three-mirror laser cavity.

The CW laser operation characteristics are shown in Fig. 2. In the plano-plano laser cavity, the laser threshold of absorbed pump power was about 0.94 W as shown in Fig. 2(a). At the common absorbed pump power of 6.12 W, the obtained maximum output powers were 1.94 and 2.58 W for output coupler transmission with 5% and 10%, respectively. Above the absorbed pump power of 5.3 W, there’s saturation effect for ceramic lasers both with T = 5% and 10%. A maximum slope efficiency as high as 61.7% was obtained by using the output couplers with T = 10%, corresponding to the optical-to-optical conversion efficiency of 42.1%. The slope efficiency decreased for lower output coupler transmission due to the lower cavity extraction efficiency and was estimated to be 44.7% for T = 5%. According to the Caird method^22^, the cavity round-trip loss was calculated to be around 6.16% by measuring the slope efficiency versus the output coupling. This loss mainly included waveguide coupling loss, propagation loss and scattering loss at the air gap. The laser exhibited multimode operation in the vertical direction due to the large thickness of the gain layer. Figure 2(c) shows the obtained beam profile in the near field, illustrating the multimode characteristics. In contrast to the well maintained Gaussian profile in the non-guided direction, the spatial profile in the guided direction was controlled by the index guiding and shows significant intensity variation across the beam. Since the three-mirror cavity possessed a more stable configuration, the laser could emit more efficient output than the plano-plano laser, as shown in Fig. 2(b). Meanwhile, increasing the cavity length decreased the confine effect of the waveguide and operate the laser in single mode as displayed in Fig. 2 (d). The best laser performance was obtained with the same output coupler of T = 10%, giving a saturated absorbed pump power of 6.5 W and a maximum output power of 3.02 W. The corresponding slope and optical-to-optical conversion efficiency were 66.0% and 46.4%, respectively. Under the same incident pump power, the maximum output power with T = 5% was 2.51 W, corresponding to a slope efficiency of 54.7%.

### Wavelength tunability properties

**Figure 3:**
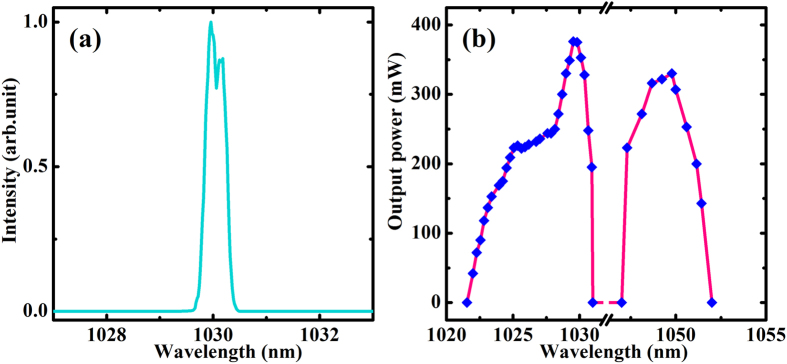
Wavelength properties. (**a**) The typical output spectrum for CW laser operations. (**b**) The tuning curve obtained with an intra-cavity dispersive prism.

Figure 3(a) shows the output spectrum measured by an optical spectrum analyzer with a resolution of 0.02 nm. The central emission wavelength located at 1030 nm and exhibited no observed changes with different output couplers, which corresponded to the two major emission bands of Yb^3+^ ion from the lowest levels of ^2^F_5/2_ to the highest level of ^2^F_7/2_ manifold. With the help of a dispersive prism, the tunable range of the planar waveguide ceramic was measured. As shown in Fig. 3(b), two tunable bands were observed for the sample. The full-width at half-maximum was 7 nm around 1030 nm and 4 nm around 1049 nm, respectively. Continuously tuning band could be obtained by optimizing the chromatic dispersion induced by the prism and cavity losses to overcome the gain competition^15^. Based on the results above, we can see that although the total tuning range herein kept comparable level with the previous results obtained by bulk ceramic with the same Yb-doping concentration^23^, this YAG/Yb:YAG/YAG planar waveguide ceramic demonstrated lower laser threshold and improved pump efficiency due to the long pump-intersection lengths in high intensity and the optical confinement for transverse mode.

### Mode-locking properties

**Figure 4:**
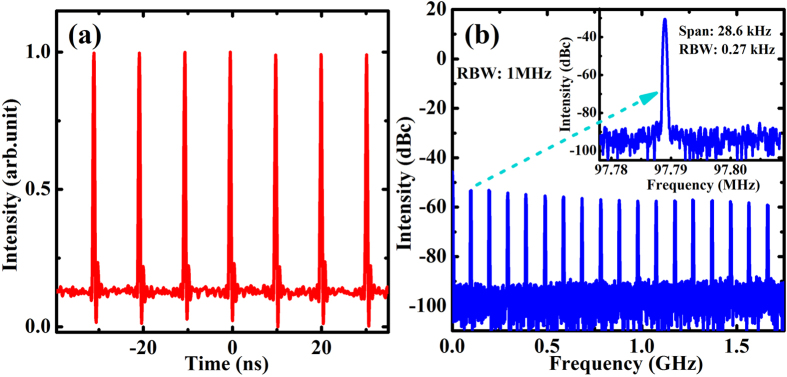
Characterization of the mode-locked YAG/Yb:YAG/YAG waveguide ceramic laser. (**a**) Recorded digital pulse trace. (**b**) Wide span radio frequency spectrum of the pulse train and zoom into the fundamental signal with 0.27 kHz resolution (inset); RBW: resolution bandwidth.

In the mode-locking operation, we carefully optimized the Z-fold cavity for clean and pedestal-free pulses with stable output in the fundamental mode. The distance between M_1_ and M_2_ was about 253 mm, while M_2_ and output coupler were separated by 540 mm, and the length between M_3_ and SESAM was 153 mm. The total cavity length was about 1530 mm. Calculated by the ABCD matrix formalism, the laser cavity got a diameter of 102 μm on the waveguide and 60 μm on the SESAM, respectively. Figure 4(a) reveals the output pulse train of the SESAM mode-locked ceramic waveguide laser, which was detected by a 1.5-GHz photodiode detector (Newport, 818-BB-30A). The mode-locked laser presented a pulse-to-pulse amplitude fluctuation in 1 ms of less than 1% in a 1-GHz digital oscilloscope (Agilent, 54833A DSO), and its radio frequency spectrum was measured using a radio frequency spectrum analyzer (Agilent, N9010A). As shown in Fig. 4(b), the full span measurement (up to 1.7 GHz) with a RBW of 1 MHz shows equally powerful harmonics with a side-band suppression of nearly 40 dB. Meanwhile, the inset in Fig. 4(b) shows a zoom into the repetition rate at 97.79 MHz with a high signal-to-noise ratio up to 60 dB, indicating the stable and reliable single-pulse mode-locked operation.

**Figure 5:**
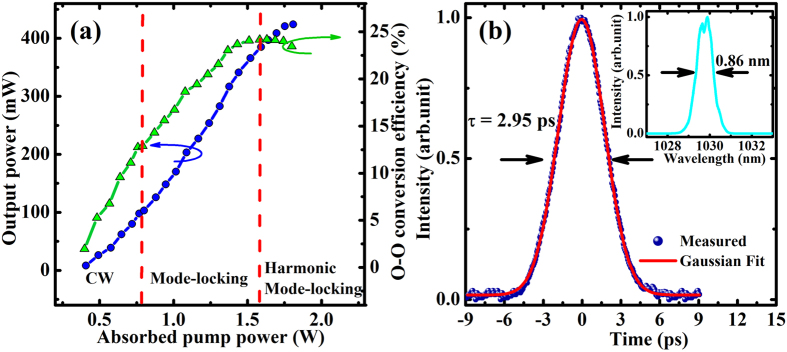
Output power properties in three laser regimes and time duration of the mode-locking laser (**a**) Output power (blue circles, left axis) and optical-to-optical conversion efficiency (green triangles, right axis) versus absorbed pump power using an OC of T = 5% in the Z-fold cavity. (**b**) Autocorrelation trace and Gaussian fit of the mode-locked laser with output power of 385 mW. Inset, the corresponding optical spectrum on a linear scale.

Figure 5(a) shows the output power and optical-to-optical conversion efficiency as a function of the absorbed pump power. The mode-locked laser threshold of the absorbed pump power was just 0.77 W with the output coupler of T = 5%. The maximum output power of CW mode-locked laser reached 385 mW with an optical-to-optical conversion efficiency of 24.2%. However, a further increase of launched pump power would lead to the harmonic mode-locking and even multi-pulse operation due to the strong energy intensity on the SESAM. By using a commercial second-harmonic intensity autocorrelator, we analyzed the time duration of the CW mode-locked pulses at the highest output power, which exhibited a pulse duration of 2.95 ps if a Gaussian-pulse shape was assumed. The inset of Fig. 5(b) shows the measured spectrum with an FWHM bandwidth of 0.86 nm at the central wavelength of 1030 nm, which supported a transform limited Gaussian-pulse of 1.8 ps.

**Figure 6:**
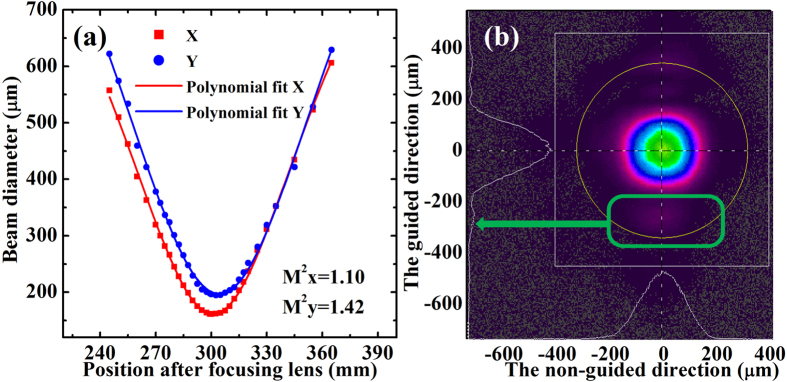
Typical beam quality factors of the mode-locked waveguide ceramic laser under the absorbed pump power of 1.59 W. (**a**) Beam diameter versus the axial distance from the focal plane of the reference lens. (**b**) Beam profile of the spatial mode captured with a CCD camera in the near field.

As shown in Fig. 6, the beam quality factors of the CW mode-locked ceramic waveguide laser were recorded by using a laser beam analyzer (Spiricon, M^2^-200S) at the maximum output power of 385 mW. As mentioned above, the waveguide was sensitive to the mode-matching due to the index guiding. A small distance change between the end-mirror M_1_ and the sample could stimulate the multimode operation. By optimizing the spot diameter on the sample, CW mode-locked laser with single mode was generated. The beam diameter versus axial distance was depicted in Fig. 6(a), indicating the corresponding M^2^ value of 1.10 in the non-guided direction and 1.42 in the guided direction. As shown in Fig. 6(b), the beam profile in the guided direction was similar to the output of the three-mirror laser and had a little pedestal, which resulted in the little bigger M^2^ value. The relatively large near-field mode profile was mainly caused by the diffusion of Yb^3+^ ions along the thickness direction during the sintering process^16^.

## Conclusions

In summary, we report on CW and mode-locked performances of a diode-pumped non-aqueous tape casting fabricated YAG/Yb:YAG/YAG planar waveguide ceramic laser. With the output coupler of T = 10%, CW output power of 2.58 and 3.02 W corresponding to the slope efficiency of 61.7% and 66.0% were achieved based on plano-plano and three-mirror laser cavities, respectively. The total wavelength tuning range of the waveguide was demonstrated to be comparable to that of the bulk ceramic. Through the use of a SESAM, an efficient 1030 nm mode-locked pulse laser operation was demonstrated at 97.79 MHz repetition rate, generating 2.95 ps pulses with maximum average power of 385 mW and M^2^ value of 1.42 in the guided direction. These results demonstrated that the technique based on non-aqueous tape casting method is an excellent way to fabricate composite structure gain medium for planar waveguide ceramic lasers and can be furthermore applied to other waveguide materials. Ultra-compact pulse laser should be possible by mounting the saturable absorber directly on the coupling mirror^24^. The Planar waveguide ceramic, which support single-mode laser in both the CW and pulse laser operations, was expected to be fabricated with thinner guiding core or appropriate geometries in the future.

## Additional Information

**How to cite this article**: Wang, C. *et al.* Ceramic planar waveguide laser of non-aqueous tape casting fabricated YAG/Yb:YAG/YAG. *Sci. Rep.* **6**, 31289; doi: 10.1038/srep31289 (2016).
